# Taking the Concept of Citizenship in Mental Health across Countries. Reflections on Transferring Principles and Practice to Different Sociocultural Contexts

**DOI:** 10.3389/fpsyg.2017.01020

**Published:** 2017-06-21

**Authors:** Francisco José Eiroa-Orosa, Michael Rowe

**Affiliations:** ^1^Yale Program for Recovery and Community Health, Department of Psychiatry, Yale School of Medicine, Yale University, New HavenCT, United States; ^2^Section of Personality, Evaluation and Psychological Treatment, Department of Clinical Psychology and Psychobiology, Institute of Neurosciences, School of Psychology, University of Barcelona, BarcelonaSpain

**Keywords:** transference, citizenship, public mental health, user involvement, recovery, mental health reform, Catalonia

## Abstract

Transferring principles and practices to different sociocultural and professional contexts in the field of mental health can be very complex. Previous research on public health policy points to difficulties in different areas such as the understanding the new concepts, their applicability in different health systems, and suitable approaches to its effective implementation. The purpose of this article is to describe and analyze the process of transferring the concept of Citizenship, from its United States origins in mental health outreach work with persons who are homeless to Catalonia, Spain. We define Citizenship as promoting the rights, responsibilities, roles, resources and relationships of persons with mental illnesses, along with a sense of belonging that is validated by other citizens. The process of this transition involves embedding Citizenship in the mental health “first-person” (internationally known as Consumer/Survivor/Peer) movement in Catalonia. The paper includes a discussion of the concept of transference, including a case example of the adoption of the concept of mental health recovery in different countries. Following this, we describe the United States Citizenship model and key elements of its development. We then turn to Spain and the evolution of its mental health system, and then to Catalonia for a brief case history of transference of the principles and practices of Citizenship to that region. The “take home message” of this work is that concepts being brought from one sociocultural and national context to another, must focus on contextualization in the ‘adoptee’s’ practices, including the balance between personal involvement and professional rigor, the involvement of key actors, and ongoing evaluation of actions taken.

## Introduction: Principles and Practice Transfer Between Contexts and Regions

Transferring innovative principles and practices to different sociocultural contexts has often been analyzed from the policy transfer and mobility perspectives, i.e., the scientific study of the implementation of public policies in places not of their origin (Stone, 2012; Cook, 2015). Similar methods have been employed to study the transfer of health systems strengthening strategies (El-Jardali et al., 2014), general (Simms, 2010) and mental health reform toward patient-centered services (Hickie et al., 2014). Previous research points to difficulties during the process of transference in areas such as the understanding of novel concepts, their applicability in a different environment, and the most suitable strategies and approaches to their effective implementation (Tervonen-Gonçalves and Lehto, 2004; Simms, 2010). In addition, resistance to and lack of motivation for change often confronts systems reformers who introduce novel concepts in different settings (Saraceno et al., 2007; Makhashvili and van Voren, 2013).

Less discussed in the literature and practice of adapting innovations to different sociocultural settings is the distinction between ‘top down’ administrative-directed change (Tervonen-Gonçalves and Lehto, 2004) and ‘bottom up’ or street-level change (Rowe et al., 1998) seen in cross-national transformation (Ovuga et al., 2007). When we talk about bottom-up approaches, we talk of processes in which users or practitioners have initiated actions to try to promote change. When we talk about top-down approaches, we refer to actions in which policymakers or health managers have initiated change. These approaches include differences in the motivation for change that the diverse actors have, and in the ways and directions in which change occurs. Processes by which policymakers and managers decide to borrow health concepts, practices, and policies of another, usually more developed, country have little to do with processes by which citizen and professional mobilizations occur. Top-down changes are guided by the interests of policy-makers and managers, which do not always coincide with collective aspirations, while bottom-up transformations must be promoted and accepted by service users and practitioners. For example, the profound influence of Paulo Freire’s *Pedagogy of the Oppressed* on the internationalization of the empowerment concept, can be understood from the critical insights that professionals borrowed for their own work. Particularly, that inequalities in access to health and education services had their origin in social inequalities and could be changed through collective action (Campbell, 2014). On the other hand, psychiatric deinstitutionalization can be understood as a top-down transformation. While stakeholders’ protests may have brought the need into public view, the ‘heavy lifted’ was done by administrators whose distance from the issues in play may partly explain some of its failures, including the increase of the homeless population with mental disorders (Lamb, 1984) and the failures of its implementation in non-Western Contexts (Shek and Pietilä, 2016).

Another case example relevant to our theme is the implementation and transference of the Recovery model, essentially adopted by the Clinton administration in the United States in 1999 with the Surgeon General’s Report on Mental Health (Davidson et al., 2006). The recovery model emphasizes the participation of consumers and their families in service planning and the promotion of recovery not as a clinical fact or occurrence but as a process of change through which individuals improve their health and wellness, live self-directed lives, and strive to reach their full potential (Substance Abuse and Mental Health Services Administration, 2012). The introduction of this model in the United States has had a clear impact on the policy and practice of mental health care. Perhaps its signal accomplishment and characteristic is to advocate for and support the participation of service users as “peer specialists” or “peer mentors” for persons with mental health disorders, and for their participation in systems reform (Davidson, 2016).

The transfer of the recovery concept and its derived practices soon spread to the rest of the Anglo-Saxon world, especially the United Kingdom, New Zealand, Australia, and Canada, as well as other developed countries (Ramon et al., 2007). Although to our knowledge there is no specific literature that analyzes the strategy of transfer of the movement toward Recovery as a public policy, we know that the consumer movement was strongly involved in protesting current practice and advocacy for change, along with academic, researchers, policy makers, and practitioners. The advocacy activities carried out by these groups and their ability to connect internationally contributed mightily to its dissemination and adoption around the world. Small-scale implementation strategies have included “top-down” activities such as training and organizational change activities for professionals (Mabe et al., 2016), sometimes provided by people with lived experience of mental disorder (e.g., Simpson, 2002), and bottom-up participatory processes such as involving service users in the design of Recovery-in-Action initiatives (Park et al., 2014).

Although the international expansion of the Recovery movement has been instrumental in deepening the processes of extension of community rehabilitation practices that, in retrospect, could have enhanced the process and outcome of psychiatric deinstitutionalization, the “colonization” of its vocabulary by policymakers has been criticized, even leading to movements such as *Recovery in the Bin*. Service users in the recovery movement, and others, have accused policy makers of harnessing the values of empowerment and mutual support that are part of the recovery movement and message to cut services and blame people for their mental ailments (Thomas, 2016). For these reasons, critical analyses aiming to support the autonomy, empowerment, and full citizenship of people with mental health problems remain a topic of discussion.

The purpose of this article is to describe and analyze the preliminary process of transferring the concept of Citizenship, a novel approach to the social inclusion and community membership of people with mental health problems, from the United States to other countries. We employ a case example of a Citizenship project developed in Catalonia, Spain as what we hope we will also serve as an instructive example of the challenges and possibilities for dissemination of the Citizenship model internationally.

## Citizenship: A Novel Approach to Mental Health

Citizenship, involves the strength of people’s connections to the rights, responsibilities, roles, resources and relationships that society offers to people through public and social institutions (Rowe et al., 2001), and a sense of belonging as full, participating members in society that is validated by one’s fellow citizens (Rowe, 2015). The strengths and limitations of outreach work, including the finding that helping people find housing does not, in itself, lead to their community integration or full membership, led a group of scholars and practitioners based at Yale to develop the theoretical framework of Citizenship (Rowe and Pelletier, 2012). Although the Citizenship framework was developed during the period of the Recovery movement (Slade et al., 2014), and analyses show that higher levels of Recovery are usually found in persons who are better able to exercise their rights as full citizens (Pelletier et al., 2015), the two approaches evolved independently.

The first Citizenship intervention was the Citizens Project, implemented at the Yale Program for Recovery and Community Health. The concept of Citizenship (Rowe et al., 2001) was used as a framework (Rowe et al., 2009) for opening up opportunities for social participation to members of stigmatized groups. In this program, rather than viewing individuals with mental illness as problems to be addressed through the intervention of others, participants are “students” and “citizens.” Hence, they are viewed as experts on many of their own problems and difficulties, on identifying solutions to them, and who are capable of learning not only how to maintain themselves stably in their communities but to see themselves as and take actions to become valued members of their communities. Citizens Project participants are persons with mental illnesses including, for many, the dual disorder of substance misuse, and previous criminal charges. A randomized clinical trial comparing the Citizenship intervention to usual care for the target group showed that it successfully reduced alcohol and other substance use, and increased quality of life for participants (Clayton et al., 2013). Following this study, participatory action research methods including peers (service users) as researchers were employed to develop an individual measure of Citizenship (Rowe et al., 2012) with seven dimensions of Citizenship—Personal Responsibilities, Government and Infrastructure, Caring for Others in Community, Civil Rights, Legal Rights, Choices and Stewardship. This measure has also been validated for use with persons with mental illness receiving public care (O’Connell et al., 2017).

The use of Citizenship as a psychosocial intervention rooted in collaborative work with and among people affected by mental health problems is related to other similar conceptualizations. A meeting point between all these conceptualizations is that Citizenship should be thought as negotiated and enacted rather than given (Stevenson et al., 2015). For example, Barnes et al. (2004) link Citizenship with the dynamics of membership and its legitimacy. They show how the entitlements associated with the category citizen are embedded in the dynamics of inclusion and exclusion of rights. Continuing with the idea of Citizenship as the legitimation of rights entitlement, and adding a transformative dimension, Renedo and Marston (2015) developed the concept of participatory Citizenship in the context of patient and public involvement in the healthcare system. They propose a dynamic view of Citizenship comprising the participation of different actors in negotiating and acting on their rights and responsibilities as health service users and drivers of change.

Similarly to our conceptualization of Citizenship, the idea of agency as opposed to prescribed interventions has emerged as an argument against pathological approaches to trauma and distress (Veronese et al., 2015). Specifically, there are lines of research that may help us understand how the structure and protocols used by mental health institutions subvert the agency of social inclusion of their clients (Watson, 2012). In general, the conceptualization of agency on which our view of Citizenship is based is systemic, with individual actions understood within the broad contexts and power relations (Diewald, 2007; Stetsenko, 2007), rather than Bandura’s (1977) conceptualization of individual beliefs of self-efficacy and Rotter’s (1954) notion of locus of control.

Our Citizenship framework is now being adapted for use in other countries and sociocultural contexts. Current Citizenship research and practice is being undertaken in Quebec (Canada), Scotland (United Kingdom) and more recently, Catalonia (Spain). After contextualizing our work within the Spanish mental health system evolution, we will discuss some of the implications of transferring the concept of Citizenship in mental health to the latter territory.

## The Spanish Case of Mental Health System Evolution and Stakeholders’ Involvement

Spain has had some differences with respect to other Western European countries in terms of the implementation of the last two major waves of mental health care reform, namely deinstitutionalization and recovery. Due to the duration of Franco’s fascist dictatorship, the deinstitutionalization of the psychiatric system did not begin until the 1980s, when alternative resources to the great psychiatric hospitals and cooperation with primary care began to be developed (Vázquez-Barquero and García, 1999). The evolution of structural changes in the Spanish Public Mental Health system has been marked by the division of Spanish psychiatry into two major professional associations: The Spanish Society of Psychiatry (*Sociedad Española de Psiquiatría*, SEP, biologically oriented) and The Spanish Association of Neuropsychiatry (*Asociación Española de Neuropsiquiatría*, AEN, community and psychotherapy-oriented). While the latter has worked together with other professional bodies (e.g., the Spanish Federation of Psychosocial Rehabilitation, *Federación Española de Rehabilitación Psicosocial* in Spanish, FEARP) and consumer-led (relatives and more recently, “first-person” consumers’) to support the implementation of community-based resources, the former has always had an ambiguous role marked by its conflicts of interest with the pharmaceutical industry (Escobar, 2009).

The arrival of the Recovery movement to Spain has been limited to community rehabilitation services and its extension to other care settings occurred only recently through specific and somewhat isolated projects. In 2005, for example, the European-wide EMILIA (acronym for *Empowerment of Mental Illness Service Users: Life Long Learning, Integration and Action*) project (Ramon et al., 2011) was one of the first to systematize joint training actions between professionals, users and their families. This project, together with community-oriented professional bodies such as the AEN and FEARP, served as an outpost of the Recovery movement in Spain. However, the arrival of the crisis in 2008 and the cuts in funding for public health that entailed led to a total withdrawal of funding for this type of projects, as the Spanish public mental health system retreated to “survival” funding for mental health care. Based on preliminary results of focus groups carried out as part of this Citizenship transfer project (see ‘Methodological Implications’ section), mental health professionals agree that in recent years they have barely been able to provide minimum services to users, who must endure long waiting lists for care. Many professionals say that this was not the time to implement “any kind of novelty,” as they are simply overwhelmed. Over the same time period there has been an intense media debate on the influence of the pharmaceutical industry, as the primary care protocols for the treatment of the increased anxiety-depressive symptoms reported in these services, caused by the economic difficulties of the population (Gili et al., 2013), have mainly involved use of antidepressants and anxiolytics.

An exception to the shortage of mental health care resources has been the incorporation of individualized monitoring programs (Programas de Seguimiento Individualizados, PSIs) staffed by professionals who provide care in community environments. Although these practitioners are not fully trained in Recovery principles, many of them are familiar with its practices. Furthermore, elements derived from other orientations such as Community Assertive Treatment share some principles with those of Recovery. Yet, as mentioned above, the Recovery approach has had a very narrow scope for practice, being confined to community rehabilitation services. The implementation of Recovery principles in places which are more difficult to influence such as hospitalization services, is still almost non-existent in Spain.

An ominous event regarding the potential to implement the Citizenship framework in Spain was the attempt to introduce “special security measures” for people diagnosed with mental disorders in the Spanish Penal Code by the conservative government that took office in late 2011. This 2013 bill produced an immediate and massive reaction from mental health advocacy groups. The campaign against the bill revived old synergies among groups that had been relatively inactive for a few years, giving additional support to the first-person (consumers/survivors/peer) movement. In Spain, this movement is formed by associations that operate in diverse contexts, from activism and policy reform to leisure and art. The Spanish Federation of Relatives of Persons with Mental Illness changed its name to “Confederation Mental Health Spain,”^1^ intending to integrate first-person organizations within the same structure. However, many organizations believed that the best approach was to construct differentiated spaces for families and those who have lived the experience themselves. A first-person federation already existed in Andalusia since 2009 and a new one was created in Catalonia in 2014. Less populated communities such as Asturias, Balearic Islands, Canary Islands, Madrid, Navarra, or Valencia have active associations. A negotiation to create a Spanish-wide federation is in process.

Andalusia and Catalonia are territories especially active in the progressive approaches to mental health care and supports because of (1) having federations that exercise the institutional representation of people with lived experience of mental disorders (“In first person,”^2^ *En primera persona* in Spanish, and “Voices,” *Veus*^3^ in Catalan, respectively), independently of relative’s and users’ associations’ federations (Andalusian Federation of Relatives and People with Mental Illness^4^, and Mental Health Catalonia^5^), and 2) having territory-specific anti-stigma campaigns. The executive boards of these campaigns are formed by relatives’ and users’ first-person federations, and by representatives of public and private mental health service providers and local governments. The Andalusian campaign is called *1 de cada 4*^6^ (“one in four,” referring to the fact that 25% of the world’s population will suffer from a mental disorder during their lifetimes) and the Catalan, “Obertament”^7^ (“openly”). In addition to its activities to prevent stigma, the “1 de cada 4” campaign has translated materials and openly supports the implementation of the Recovery paradigm in the Andalusian public mental health care system. Obertament, for its part, trains first-person activists to carry most of their activities, and is designing a campaign to educate and influence health care professionals. In the case of Catalonia, as a result of the collaboration of both federations, a project has recently been launched (*Activa’t per la salut mental*, ^8^ literally “get involved in mental health”) which aims to promote the recovery model through information, psychoeducation, empowerment and mutual support spaces.

Having reappeared under the threat of hardening of the penal code for persons diagnosed with a mental health disorder, the “first-person” movement made the struggle for rights a core objective of its work. At the same time, the institutional anti-stigma “1decada4” and Obertament campaigns, and the relatives’ movement also supported prioritizing defense of the rights of people diagnosed with mental health problems.

## From Big Concepts to Daily Work Within the Catalan Mental Health Network

**FIGURE 1:**
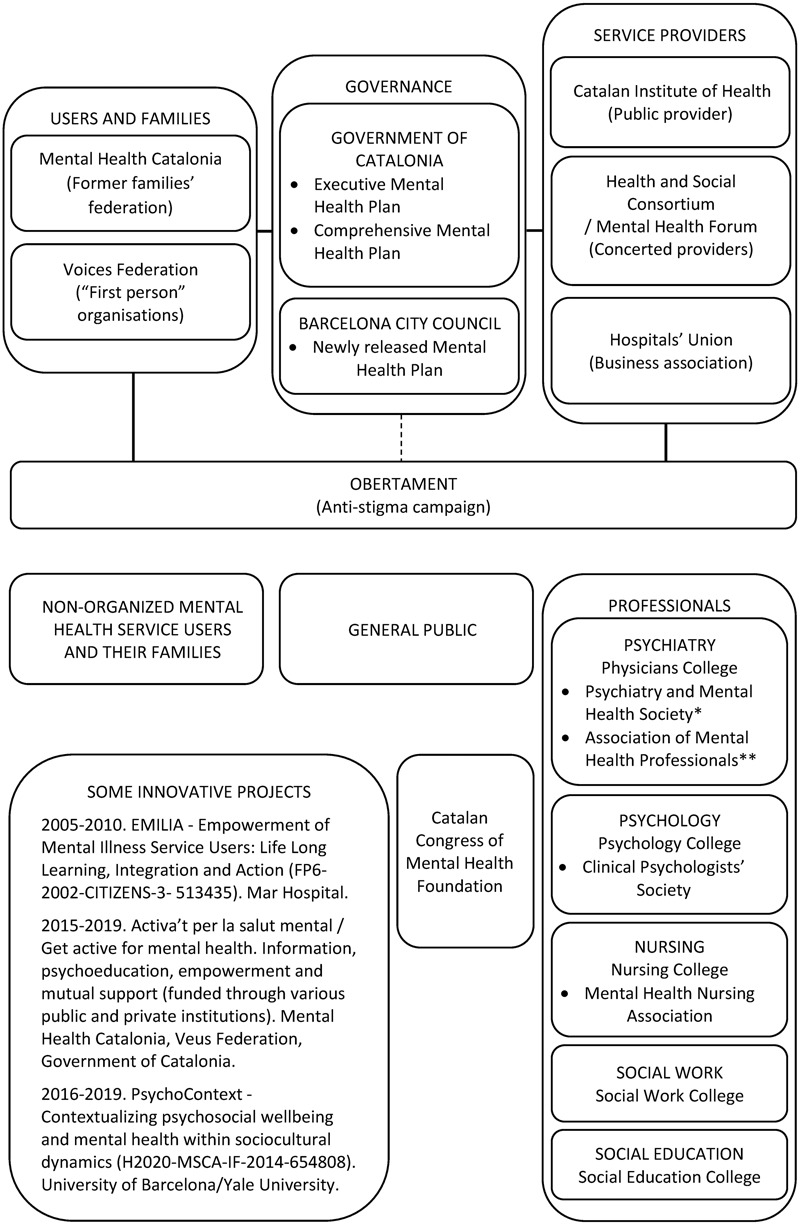
Principal Mental Health Stakeholders in Catalonia. ^∗^The Catalan Psychiatry and Mental Health Society is related to the Spanish Society of Psychiatry, of neurobiological orientation, although it is not an organic part of it. ^∗∗^The Association of Mental Health Professionals is the local branch of the Spanish Association of Neuropsychiatry, with psychotherapeutic and community orientation, and admits the participation of psychologists, nurses, social workers, educators and other professionals. It has recently begun to welcome the debate on the affiliation of service users.

**Figure 1** shows the structure of the Catalan mental health network. As can be seen at the top of the figure, there are three main political representation groups. The local administration promotes executive (centered in the health system) and comprehensive (coordinated with other departments such as work or justice) mental health plans. Additionally, the city of Barcelona has launched its first local mental health plan. In collaboration with these administrations, users’ and relatives’ representatives collaborate with service providers in the governance of the *Obertament* campaign against stigma.

At the bottom of the figure we see the three groups receiving messages from the Obertament campaign, and the remainder of activities involving the implementation and improvement of mental health services and societal wellbeing provided by users’ and relatives’ federations, local administration and service providers. Their messages are obviously not always univocal. While unorganized service users, families and society in general receive these messages in passively, it is important to note that mental health professionals have complex organizational structures independent of the service providers’ representation structures. In addition to the professional colleges of all direct care professions, there are specific professional associations in the cases of psychiatry, clinical psychology and mental health nursing. In Catalonia, there are two associations of psychiatry with different philosophical orientations regarding mental health intervention. The Catalan Association of Mental Health Professionals, as the local branch of the Spanish Association of Neuropsychiatry. The latter accepts the participation of psychologists, nurses, social workers, educators and other direct care professionals, as well as service users and their relatives. The Catalan Society of Psychiatry and Mental Health, without being a local headquarters of the Spanish Society of Psychiatry, shares some of its scientific values, framed in a biomedical vision of mental disorders. In addition, the Catalan Congress of Mental Health Foundation, which emerged from the organization of an annual congress, brings together different professionals with a focus on human rights.

In this context, and inspired by the model of Citizenship originated in New Haven, the first author of this article began his journey as an agent of change of the Catalan Mental Health system in mid-2015. In the first place, his positioning regarding participation in participative spaces both for professionals and service users was considered important. As an academic and certified psychotherapist, he contacted some of the aforementioned professional bodies, which showed some interest and invited him to give lectures to explain the project in public, with the understanding that more concrete activities must follow. Examples of these are training and educational activities, the design of which will be developed at the same time as the project itself. As a former mental health service user, the first author initiated activist training for the *Obertament* campaign in Barcelona. The involvement of the “first-person” associations in this campaign facilitated his contact with the self-managed movement, where he was recognized as a first-person participant like others, a fact that facilitated the process. In addition, his recognition and inclusion as a mental health academic provided a source of additional human capital, and he soon became involved in tasks where his academic skills were useful.

As we have said before, the struggle for the rights of people diagnosed with a mental disorder is a key objective of the first-person mental health movement. Thus, a key strategic action for incorporating Citizenship into the spectrum of mental health in Catalonia was participation in a campaign to reduce or eliminate mechanical restraints in mental health units.

The other two strategic actions that have been considered appropriate to foster within the present project and that match the objectives of other users’, families,’ and professionals’ groups, are training and hiring peer support staff and carrying out awareness activities and training for professionals. In this regard, some of the associations within the Veus federation already provide training to moderators of mutual support groups and perform different awareness-raising activities for mental health professionals, including a yearly lecture series for all mental health trainees in Catalonia. It is understood that these activities must be enhanced to make them accessible to all professionals, regardless of their or their institutions,’ philosophical and practice orientation. In a context of partial implementation of the recovery approach, the incorporation of peer-support staff only makes sense if mental health professionals are motivated to accept staff with lived experience of a mental disorder. As such, we hope to learn not only from the successes but also the challenges of countries such as the United States that has been implementing recovery-oriented policies for the last two decades (Davidson, 2016). Finally, people offering peer support need to be able to conduct other activities, such as helping people navigate the mental health system including psychiatric hospitals.

### Methodological Implications

Our project to transfer practices and approaches based on Citizenship is built on strong personal and professional involvement and a strategy with concrete objectives. Hence, although it has the strength of close collaboration and personal involvement within the main stakeholders involved, it is critical that we also maintain scientific rigor in evaluating all interventions carried out through this initiative.

**FIGURE 2:**
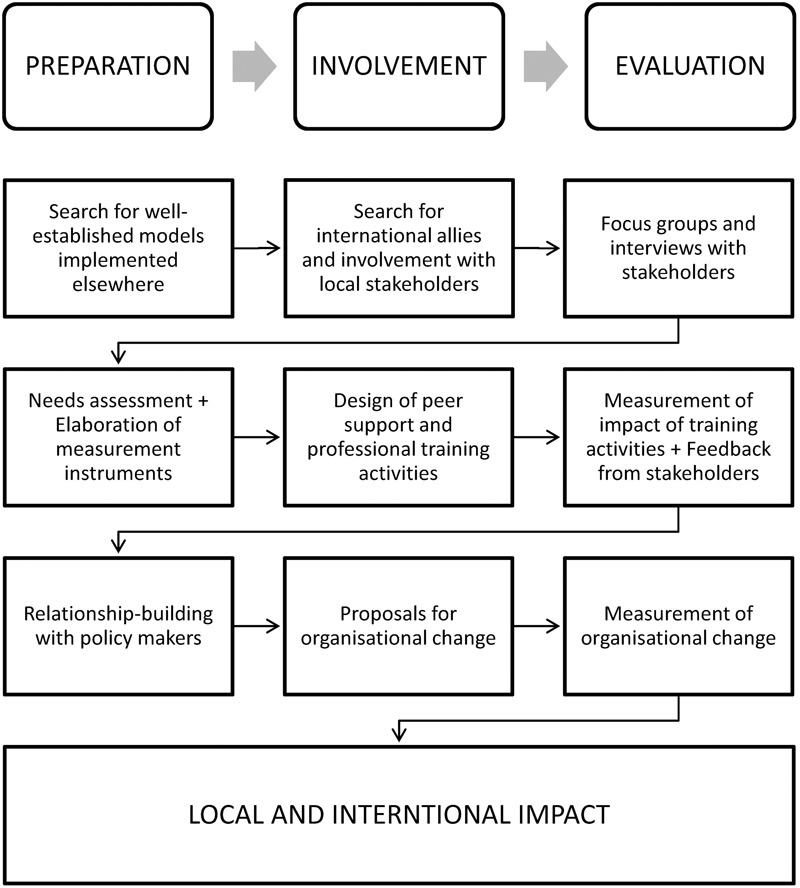
Proposed cycle of development of a transference project within a mental health system.

In general terms, starting from the theoretical elaboration of the sociocultural dynamics of well-being and distress, our project aims to promote Citizenship practices through continuous training of professionals and users of mental health services and their relatives. This initiative will benefit from the experience of the Yale program, but must also attend to the characteristics and priorities of local stakeholders. **Figure 2** shows a representation of a proposed cycle of development of our transference project within a complex mental health system. The three columns represent sets of tasks that are repeated cyclically: preparation, involvement, and evaluation of the results. The three states of activity boxes reflect current and ongoing work.

In the first stage, international models and allies were sought. In this phase, the first author met the second, a senior academic director of the Yale Citizens Project. Once funding and ethical approval were obtained (University of Barcelona’s ethics approval reference IRB3099), focus groups were held with local stakeholders. Fifteen focus groups including 70 mental health professionals, 40 service users and 10 relatives were conducted. Our choice of strategic actions was supported by the preliminary results of these focus groups obtained through thematic analysis (Braun and Clarke, 2006). These results show how speaking about Citizenship and its most intuitive components, rights and responsibilities, implies a change that can be facilitated by user participation in the design and implementation of interventions. **Table 1** shows selected quotes from interviewees supporting the need for self-determination, one of the main themes arising from the analysis.

**Table 1:** Quotations related to self-determination themes.

Service users	*“For me being a citizen means being able to express myself freely and without any fear: To be able to use my principles and my ideas and express my needs, feelings…”.*
	*“In my process one of the most important moments, the one I remember with most affection, it is when a friend, who had never been diagnosed, told me that he had also had a bad moment and told me how he bounced forward doing things with his friends, keeping himself alive. Feeling that someone understands you as an equal.”*

Relatives of service users	*“So, for you to be able to make decisions about your own life you should be totally autonomous, right? Because making decisions about your own life when you depend on another person is a bit relative.”*
	*“So, I think that the tendency now is to try to make the person who has the disorder have their own autonomy, their own life, dispose, make, and have their own rights.”*

Mental health professionals	*“I believe that the human right that is least protected is the right to decide. I think the professionals still decide for them: in an office, in the white coat, we inform the decision... But we do not have direct contact with the person.”*
	*“Talking about a decision, about their life, they ask you: “How do you see it?” Or “I’ll talk to my mother to see what she thinks” or “I’ll talk to my psychiatrist to see what she thinks.” “No, no, but... What do you want?” I think we have to work this issue a lot, from empowerment, right?”*

Once this exploratory phase was completed, needs assessments were carried out and valid measurement instruments were designed to evaluate activities of mutual support and training of professionals. The proper evaluation of these activities has been a source of arduous discussions. The use of controlled experimental designs entails the exclusion of some users and professionals from training activities, due to their disagreement with the fact of starting a process in which they may end up in a control group. Therefore, we have decided to evaluate all our activities, where possible, through wait-list-controlled randomized experimental designs. Thus, all participants complete a baseline evaluation. Only half of these (selected randomly) have immediate access to the training activity. Nevertheless, once the first follow-up has been carried (approximately 1 month after the activity is completed) the second half can be involved in the activity.

Wait-list-controlled randomized experimental designs allow for two very important flexibility features. The first is the possibility of randomizing individuals or blocks, as in some cases activities must be offered to whole clinical teams or service user groups, but in others, individual participation is more appropriate. The second is that it is not necessary to wait until all the participants have been included to carry out the randomization. Rather, blocks of participants based on natural groupings may be included until the capacity of two training activities is completed. Thus, an activity will take place immediately after enrollment and randomization and the same activity will take place a month later, for participants who were randomized “out” at baseline. This also allows for evaluation of possible biased expectations on the part of different participant groups since, once the activity has been carried out, the changes in the evaluation measures should be identical both in the first group that carried out the activity and among the members of the waiting list group.

The final project phase includes an intense activity to interact with a diverse range of health managers and policy makers with the intention of proposing profound organizational changes. This phase, however, only makes sense if at the same time, we are offering stakeholders’ feedback, in the form of scientific evaluation of their activities, and carrying out training and awareness activities.

### Engagement and Implications of Strategic Activities

All the tasks described above involve the professional and personal involvement of many people, beyond professionals and volunteers involved in these changes. They also involve the resolution of complex conflicts and the careful choice of allies. In our case, as university faculty, doing such a job involves maintaining the balance between the requirements of an academic institution and the objectives of the first-person movement. The former is focused on finding meaningful results from rigorous evaluation and publication in prestigious peer-reviewed journals. The latter is focused on physical presence, personal relationships, and constancy in the struggle, a militancy that must go beyond standard professional commitment, as the potential interpersonal conflicts that can occur in the context of these struggles are often related to intimate and sometimes traumatic experiences.

Despite the prioritization of implementation projects including training and awareness activities facilitated by funding that had been absent during the 1st years of crisis, the need to build bridges with local policy makers is a key activity. Of the three projects mentioned (mechanical restraint reduction, peer support and training/awareness activities for professionals), the reduction of mechanical restraints requires the greatest amount of collaboration with, and support from policy makers, including meetings and spaces where all voices, including those of both service users and professionals, will be heard. Thus, following a series of meetings with the director of the Executive Plan of Mental Health and Addictions in 2016, this project has been finally articulated through the Service of the Promotion of Patient Safety of the Government of Catalonia’s Department of Health, which also includes the reduction of mechanical restraints in minors and older adults in different restrictive contexts. Recently, the Veus Federation of mental health entities in first person appeared before the Health Commission of the Parliament of Catalonia (2017) to explain this project, which aims to eliminate mechanical restraints in all mental health care facilities in Catalonia before 2025. The first author of this article elaborated a collection of arguments in favor of the elimination of these coercive practices that was delivered to the deputies of the commission (Eiroa-Orosa, 2017).

The second strategic action that would benefit from institutional support is peer support training and implementation, involving funding to make this work possible and the institutional commitment this funding would represent for subsequent recruitment of trainees. A plan for peer support incorporation in the mental health workforce is included in the Executive Mental Health Plan Working Groups for the period 2017–2020. However, given the experience of the EMILIA project, which has advocated to establish peer support training for a decade, it has been considered important to develop actions without explicit institutional support. As such, workshops have been developed with the collaboration of relatives and professionals for the training of “mutual support technicians,” in collaboration with the Mar University Hospital. Furthermore, the Veus federation, of which EMILIA is part, supports the development of self-managed spaces for peer support training.

Regarding the third strategic action—training and awareness-raising activities—practitioner associations in charge of training residents in psychiatry, forensic medicine, clinical psychology and nursing (see **Figure 1**), have included these activities in response to advocacy by the federations of Catalan relatives’ and service user associations. Additionally, funding has been achieved by the Veus federation and its associations to offer a training activity called: *“Beyond Recovery: toward a rights-based mental health care.”* This activity will be offered to mental health professionals regardless of their level of experience, orientation or place of work.

While this prioritization of activities has been undertaken with consideration of the interests of key stakeholders, the adequacy of the Citizenship framework has become evident. In all these strategic actions, the importance of the main components of citizenship—rights, responsibilities, roles, resources and relationships—is incorporated to show that full recovery is not possible if people are not considered full citizens with the right to choose their own way. The aim is to enhance the rights of people with mental health disorders through increasing the responsibility they have over their lives, allowing them to take on meaningful roles and helping them gain access to resources that will enable them to be full and valued members of their communities and society in conditions of equality.

Although the differences between the systems of support for people with mental health problems between the United States and European countries with a public health system go beyond the scope of this article, we consider it fundamental to discuss the implications that these differences may have on a process of conceptual and practice transfer. The existence of a purely public and universal system of health care in a country such as Spain ensures that all residents in the territory receive adequate attention, with a life expectancy of 83 compared to 79 in the United States (Kontis et al., 2017). This said, the possibilities of innovation in a highly bureaucratized system in serious financial crisis, as is the case in Spain, are lower. This applies, for example, to arduous efforts to hire mutual support (peer) staff, with innovation filtered through many layers of bureaucratic prioritizing and decision making. In addition, the forums in which such decisions are made, depend on the ideological orientation of political power and the ability of policymakers to release budget allocations.

One of the characteristics that facilitated the emergence of the movement toward recovery in the United States was the increase in funding made available from the closing of state hospitals, though by no means the automatic transfer of it to community services. Even so, mental health activists and reform-minded mental health professionals had the opportunity to lobby for the use of these resources for community mental health care. In Spain, reformist professionals carried out the process of deinstitutionalization with the approval of the health authorities.

Furthermore, in the 1990s the Spanish relatives’ movement began to manage a part of the psychosocial rehabilitation services. However, the first-person movement so far has not reached a level of management capacity comparable to that of the Anglo-Saxon countries. For this reason, and probably also because of the prejudices about the capacity of people with mental health problems, their voices have largely been absent when service planning decisions are made. The project of transferring models such as citizenship, we argue, will only take root if the efforts and results of our evaluations are made available for debate in public spaces with full participation and empowerment of all interest groups, including those with lived experience of mental illness.

## Take-Home Message

The beginning of the transference of a concept such as Citizenship from Connecticut in the United States to Catalonia in Spain, involves the work of academics and activists but also that of many others who have long advocated for the rights of people diagnosed with mental disorders. The creation of different synergies among first-person, relatives’ and professional organizations has been important in planning strategic actions for a successful transfer process. We think that a former service user being one of the main advocates for and actors in this initiative is important, as indicated by a growing body of literature on the contributions of mental health professionals who have experienced mental illness (Richards et al., 2016; Spaulding, 2016).

This initiative involves four key (groups of) actors—users, families, professionals and policy makers. Although the involvement of the first author is greater with the first and third group, he constantly attends meetings and provides as much as possible feedback and personal implication to the other two actors. Acting otherwise would limit the Citizenship movement to only one of the actors in this process and therefore, would have insignificant impact on the lives of users.

The overlap of Citizenship efforts with the incomplete implementation of recovery-based care in Spain has at times made it difficult for us to identify ways in which this project supports citizenship, or recovery, or both. In any case, both movements are dedicated to transforming the understanding, care of and support for people with mental health diagnoses to assure that they are the leading actors in their own recovery processes, and in their achieving full citizenship. We are convinced that both fostering Recovery and Citizenship practices are desirable. However, with an eye to criticisms of Recovery discussed earlier, this transfer process is not intended to be an exercise in neocolonialism. Our intention is not to implement an idea coming from an institution (Yale) whose prestige has obvious connotations, but to benefit from the evaluation possibilities that the process has had in a resource-rich context. The fact that the project has been initiated in a prestigious institution with access to resources and carrying a certain imprimatur, facilitates the justification and legitimacy of transfer. The use of focus groups with the groups involved as the first research tool and the professional and professional involvement of the principal investigator of the United States project, help to support the academic viability of the project in the Spanish context.

We are aware that many innovations do not occur through participatory processes such as we describe here. Our main objective is that innovations such as the Citizens Project can reach the maximum number of practitioners, with adaptation as needed for different contexts. Doing so might facilitate the extension of these concepts not only to the psychosocial rehabilitation sector, but also to other mental health care sectors that must promote and safeguard the dignity and agency of their users.

## Author Contributions

Both authors wrote and reviewed the whole paper. The content’s responsibility is shared.

## Conflict of Interest Statement

The authors declare that the research was conducted in the absence of any commercial or financial relationships that could be construed as a potential conflict of interest.

## Footnotes

1 http://consaludmental.org/

2 http://enprimerapersona.org/

3 http://veus.cat/

4 http://feafesandalucia.org/

5 http://www.salutmental.org/

6 http://www.1decada4.es/

7 http://obertament.org/

8 http://activatperlasalutmental.org/
